# Analysis of *LOXL1* polymorphisms in a United States population with pseudoexfoliation glaucoma

**Published:** 2008-01-29

**Authors:** Pratap Challa, Silke Schmidt, Yutao Liu, Xuejun Qin, Robin R. Vann, Pedro Gonzalez, R. Rand Allingham, Michael A. Hauser

**Affiliations:** 1 Department of Ophthalmology, Duke University Eye Center; 2 Center for Human Genetics, Duke University Medical Center, Durham, NC

## Abstract

**Purpose:**

To identify if recently described *LOXL1* (lysyl oxidase-like 1) polymorphisms are associated with pseudoexfoliation glaucoma (XFG) in a United States (U.S.) Caucasian patient population.

**Methods:**

Individuals with XFG were identified using standard clinical examination techniques. TaqMan allelic discrimination assays were used to genotype 13 single nucleotide polymorphisms (SNPs) that tag *LOXL1* in Caucasian individuals. The coding region of exon 1 that includes the previously associated SNP, rs1048661, was sequenced. Allele and genotype frequencies were compared between cases and unrelated controls.

**Results:**

Fifty affected individuals and 235 control individuals were recruited into this study. We replicated the previously reported association of three SNPs (rs1048661, rs2165241, and rs3825942) in our independent XFG population (single SNP p-values were 0.001-0.02). The risk alleles at these three and several other intragenic SNPs are part of an extended XFG-associated *LOXL1* haplotype with a frequency of 32.0% in XFG patients and 21.6% in controls.

**Conclusions:**

We have performed an analysis of *LOXL1* and XFG in a United States patient population and have confirmed the strong association previously reported for Icelandic and Swedish samples. However, due to the high frequency of risk alleles in non-XFG individuals, this association should not form the basis of a diagnostic test for XFG. It is likely that additional genetic or environmental factors modulate the penetrance of *LOXL1* susceptibility alleles.

## Introduction

Pseudoexfoliation syndrome (XFS) was initially described by Lindberg in 1917 [1] and further characterized by Vogt in 1925 [2]. It is a systemic disorder in which an unidentified, fibrillar substance is produced in abnormally high concentrations. The incidence of XFS varies among ethnic groups [3] with incidences of 20%–25% in the Scandinavian countries of Iceland and Finland [4] to 0% reported in Greenland Eskimos [5]. The Framingham Eye Study from the United States [6] revealed an overall incidence in non-glaucoma individuals of 0.6% at the age of 52–64 years that rose to 5.0% for 75–85-year-old individuals. Caucasians over 60 years old in the United States have an incidence of PEX around 1.6%-3% and African-Americans are approximately 0.4% [7,8].

Both Lindberg [1] and Vogt [2] noted the association of XFS with glaucoma and increasing age, and it is a major cause of glaucoma throughout the world [3,9,10]. In general, the incidence of glaucoma in XFS patients in the United States appears to vary with the individual’s ethnic background. The prevalence appears to be highest among individuals with Scandinavian ancestry and lower among African-Americans [8,11]. The reported prevalence of XFG varies among ethnic groups and ranges from 0.4%-28% of open angle glaucoma in the United States [8,11-13].

Recently, Thorleifsson et al. [14] performed a genome-wide association study and identified a strong association of XFG with three single nucleotide polymorphisms (SNPs) in the lysyl oxidase-like 1 gene (*LOXL1*). They identified one intronic SNP (rs2165241) and two nonsynonymous coding SNPs (rs3825942 and rs1048661) with significant disease association in Icelandic and Swedish individuals. This association was recently replicated in both the midwestern United States [15] and Australian [16] populations.

*LOXL1* belongs to the “LOX” family of extracellular enzymes that have multiple functions including the cross-linking of collagen and elastin by oxidatively deaminating lysine residues. Since XFS deposits are associated with the extracellular and basement membrane regions, the LOX genes are legitimate functional candidates to be involved with XFG pathogenesis [17]. There are five such enzymes (LOX, LOXL1, LOXL2, LOXL3, and LOXL4) that are involved with extracellular matrix metabolism. LOXL1 has been implicated in the pathogenesis of spontaneous cervical artery dissection [18] as well as bladder cancer [19].

## Methods

### Patient ascertainment

This study adhered to the tenets of the Declaration of Helsinki. The research protocol was approved by the Duke University Institutional Review Board, and all patients consented to participating in the study. All patients were examined by board certified glaucoma specialists.

Pseudoexfoliation changes were identified as the presence of a central disk of XFS material, a clear annular zone (partial or complete), or flakes of XFS material on the lens surface, iris, or corneal endothelium in either eye. Patients were excluded if there was a history of exposure to intense infrared light, for example, glassblowing is associated with true exfoliation of the lens capsule rather than XFS. XFG was diagnosed when patients possessed the above XFS characteristics and at least two of the following criteria: A) documented intraocular pressure (IOP) ≥22 mmHg in either eye; B) glaucomatous optic nerve cupping defined as a cup to disc ratio >0.7 in either eye, notching of the neuroretinal rim, or an asymmetric cup to disc ratio >0.2; and/or C) glaucomatous visual field loss consistent with the optic nerve appearance. Glaucoma suspects were excluded from this study. All cases and controls were Caucasian. Controls were individuals of similar age as the patients without any evidence of pseudoexfoliation deposits on intraocular tissues. Their IOPs were in the normal range (<21 mmHg) with normal-appearing optic nerves.

### Single nucleotide polymorphism selection and genotyping methods

**Figure 1:**
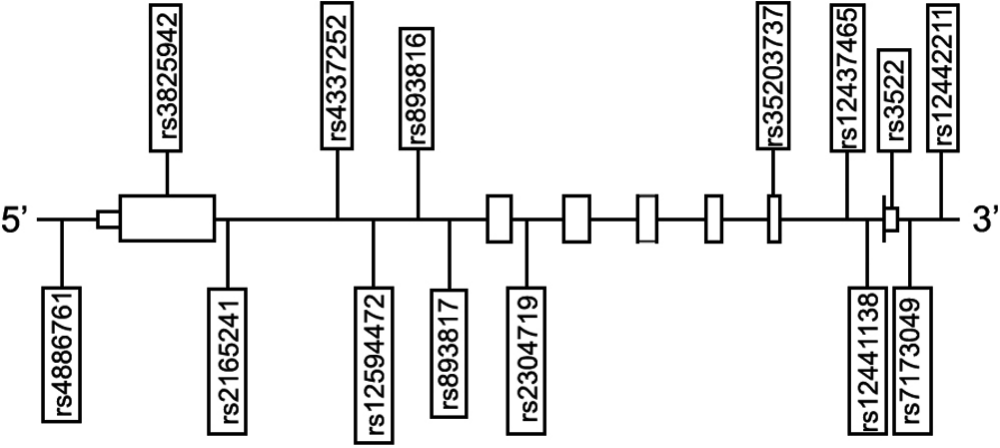
*LOXL1* gene structure with the position of each SNP as indicated

Blood samples were obtained from each individual via peripheral venipuncture, and genomic DNA was isolated using standard techniques (Gentra, Minneapolis, MN). Tagging SNPs for Caucasian individuals were selected using Tagger in Haploview 3.32 with a pairwise r^2^ threshold of 0.8, based on genotype data generated by the HapMap project. In addition to tagging SNPs, we also genotyped the three specific SNPs implicated by the previous study of XFG patients and controls [14]. See Figure 1 for *LOXL1* gene structure and relative locations of SNPs used in this study. TaqMan allelic discrimination assays were performed for all SNPs per standard protocols from the manufacturer (Applied BioSystems, Foster City, CA). Two Centre d'Étude du Polymorphisme Humain (CEPH) standards were included in each 96-well plate for quality-control (QC), and samples from six individuals were duplicated across all plates with the laboratory technicians blinded to their identities. Genotype submission to the analysis database required matching QC genotypes within and across plates and at least 95% genotyping efficiency.

Diagrammatic representation of the *LOXL1* gene. The relative positions of the 14 SNPs genotyped in this study are indicated below and demonstrate that both flanking and intragenic SNPs covering the entire gene were analyzed.

### Sequencing

All sequencing was performed using appropriately selected primers and conditions optimized in a standard fashion. The coding region of exon 1 that included the SNP, rs1048661, was sequenced because a TaqMan assay could not be designed. All sequencing was performed using an ABI 3730 capillary sequencer (Applied BioSystems).

### Statistical Analysis

Genotype frequencies of XFG cases and controls were compared by logistic regression with adjustment for age and sex using SAS software (SAS Institute Inc., Cary, NC). Analysis of Hardy–Weinberg equilibrium (HWE) was performed separately for cases and controls using GDA software [20]. As in the original study by Thorleifsson et al. [14], SNP genotypes were coded according to a multiplicative (log-additive) model in which the disease risk in carriers of two variants is assumed to be the square of the risk in carriers of a single variant. Haplotype analysis was performed with the haplo.stats package [21,22].

## Results

Fifty XFG patients and 235 control individuals were recruited into this study. All patients (39 males-78% and 11 females-22%) and controls (145 males-61.7% and 90 females-38.3%) were Caucasian. The mean age of diagnosis was 74.0 (standard deviation,SD, was 8.0) years in patients; the age at recruitment was 64.9 (SD 11.6) years for the controls. All SNPs were in HWE in cases (p>0.05) and controls (p>0.05). Table 1 presents the results of the logistic regression analysis and the linkage disequilibrium (LD) coefficients (r^2^) between each genotyped SNP and the previously implicated XFG susceptibility marker, rs3825942 (G153D). We confirmed the previously reported association between XFG and rs2165241 (p=0.001), rs1048661 (p=0.02), and rs3825942 (p=0.02). Due to inter-marker LD, several other intragenic SNPs also showed evidence of association at p≤0.02. The haplotype analysis of SNPs, rs1048661 and rs3825942, confirmed that only three of the four possible haplotypes were observed in our sample (D’=1); the haplotype formed by the two protective alleles (T, A) was not detected. However, the frequency of the high-risk haplotype (G, G) in our population was lower than in the Icelandic and Swedish populations (United States: 69.9% in XFG cases, 46.6% in controls; Iceland: 81.4% in XFG cases, 49.8% in controls; and Sweden: 83.3% in XFG cases, 56.1% in controls). A haplotype analysis of all 12 SNPs with minor allele frequency (MAF)>5% confirmed that all individually associated SNPs (p<0.05 in Table 1) were part of the same XFG-associated haplotype, which had an estimated frequency of 21.6% in controls and 32.0% in XFG patients (haplotype-specific p-value was 0.03). All other *LOXL1* haplotypes occurred at less than 10% frequency in controls. This is consistent with high pairwise D’ values between the coding SNP, rs3825942, and the other XFG-associated markers (D’>0.9) but relatively low r^2^ values (Table 1) due to different allele frequencies.

**Table 1:** Allelic association results for the 14 tagging single nucleotide polymorphisms in *LOXL1*

SNP	Physical position (bp)	Allele	Frequency (%)		OR (95% CI)	p value	r^2^ with rs3825942
cases				controls			
rs4886761	72002604	T	50.0	37.7	1.74(1.11, 2.72)	0.0159	0.067
rs1048661 **(R141L)**	72006599	G	78.7	66.5	1.86(1.10, 3.15)	0.0222	0
rs3825942 **(G153D)**	72006635	G	93.9	84.4	3.05(1.20, 7.76)	0.0194	–
rs2165241	72009255	T	66.7	48.7	2.30(1.40, 3.76)	0.001	0.133
rs4337252	72013818	G	68.4	50.7	2.30(1.40, 3.76)	0.001	0.141
rs12594472	72014193	T	3.1	2.2	1.43(0.37, 5.50)	0.6081	0.002
rs893816	72015517	C	78.6	66.4	1.89(1.12, 3.17)	0.017	0.008
rs893817	72016118	A	75.5	62.6	1.94(1.18, 3.20)	0.0096	0
rs2304719	72022553	C	82.7	71.0	2.21(1.22, 4.03)	0.0098	0.311
rs12437465	72030299	T	71.4	56.7	2.00(1.23, 3.26)	0.0055	0.163
rs12441138	72030950	A	5.1	3.6	1.63(0.56, 4.78)	0.3801	0.007
rs3522	72031397	T	42.7	40.9	1.12(0.72, 1.75)	0.6349	0.089
rs7173049	72031663	A	78.1	77.7	1.09(0.63, 1.88)	0.783	0.131
rs12442211	72032728	G	55.3	47.5	1.31(0.85, 2.03)	0.2256	0.084

p values are based on a multiplicative model of risk. The three previously implicated SNPs are shown in **bold** [14]. Linkage disequilibrium coefficients (r^2^) of all genotyped SNPs with rs3825942 are shown in the last column. OR stands for odds ratio, and CI stands for confidence interval.

The two coding SNPs, rs1048661 (R141L) and rs3825942 (G153D), were analyzed for their ability to predict affection status. The rs1048661 SNP demonstrated a 95.7% sensitivity (45 of 47 cases have the G allele) but only 13% specificity (28 of 215 controls lack the G allele) as a diagnostic test for XFG. The rs3825942 SNP demonstrated a 100% sensitivity (49 of 49 cases have the G allele) but only 3.1% specificity (7 of 225 controls lack the G allele).

## Discussion

Thorleifsson et al. [14] recently reported the results of a genome-wide association study of XFG that identified three strongly associated *LOXL1* sequence variants, two of which were nonsynonymous coding SNPs. With this study, we have replicated these associations in a U.S. population of XFG patients and controls.

However, we demonstrate that these associations are not strong enough to justify a diagnostic test for XFG. While the rs1048661 and rs3825942 SNPs individually have high sensitivity, their specificity is very poor (3.1% and 13.0%, respectively) due to their high prevalence in individuals without XFG. This same high prevalence of *LOXL1* risk alleles has been reported in all populations examined to date: Nordic [14], midwestern United States [15], and Australian [16]. This suggests that additional genes or environmental factors affect the penetrance of these *LOXL1* sequence variants. Further investigation of the complex etiology of XFG is warranted.
